# A quick convergent-beam laboratory X-ray reflectometer using a simultaneous multiple-angle dispersive geometry

**DOI:** 10.1107/S1600576717002461

**Published:** 2017-03-24

**Authors:** Wolfgang Voegeli, Chika Kamezawa, Etsuo Arakawa, Yohko F. Yano, Tetsuroh Shirasawa, Toshio Takahashi, Tadashi Matsushita

**Affiliations:** a Department of Physics, Tokyo Gakugei University, 4-1-1 Nukuikita-machi, Koganei, Tokyo 184-8501, Japan; b Photon Factory, Institute of Materials Structure Science, KEK, Tsukuba, Ibaraki 305-0801, Japan; c Department of Physics, Kinki University, Higashiosaka, Osaka 577-8502, Japan; d National Metrology Institute of Japan, National Institute of Advanced Industrial Science and Technology, Tsukuba, Ibaraki 305-8565, Japan; e JST, PRESTO, Kawaguchi, Saitama 332-0012, Japan

**Keywords:** specular X-ray reflectivity, time-resolved measurement, laboratory X-ray sources, characteristic X-rays

## Abstract

An X-ray reflectometer using a laboratory X-ray source for quick measurements of the specular X-ray reflectivity curve is presented. It uses a bent–twisted crystal to monochromatize and focus the diverging X-rays (Cu *K*α_1_) from a laboratory point source onto the sample. The reflected X-rays are recorded with a two-dimensional detector. Reflectivity curves can be measured without rotating the sample, detector or X-ray source during measurements. The instrument can separate the specularly reflected X-rays from the diffuse scattering background, so low reflectivities can be measured accurately. For a gold thin film on silicon, the reflectivity down to the order of 10^−6^ was obtained with a measurement time of 100 s and that down to 10^−5^ with a measurement time of 10 s. Reflectivity curves of a silicon wafer and a liquid ethylene glycol surface are shown as well. Time-resolved measurements of a TiO_2_ surface during UV irradiation are also reported.

## Abstract 2

An X-ray reflectometer, using a laboratory X-ray source, that can measure the specular X-ray reflectivity curve with a time resolution of 10 s or less was developed. Low reflectivities can be measured accurately because the background can be subtracted from the signal.

## 1. Introduction   

Specular X-ray reflectivity is an established tool for structural characterization of surfaces and interfaces of materials (Als-Nielsen *et al.*, 1994 ▸; Daillant & Gibaud, 1999 ▸). The most common method for measuring specular X-ray reflectivity curves is the angle-scan method, where a collimated monochromatic X-ray beam is incident on the sample surface and the reflected intensity is measured point by point, each time changing the glancing angle of the X-rays by rotating the sample and detector. This procedure for scanning the angle of the sample and detector limits the attainable time resolution. For time-resolved observation of irreversible structural changes, where the pump–probe method (Nüske *et al.*, 2011 ▸) cannot be used, the time resolution of the angle-scan method is of the order of minutes, even using synchrotron radiation (Gonzalez-Silveira *et al.*, 2007 ▸; Yano *et al.*, 2010 ▸).

The energy-dispersive method using a solid state detector and *Bremsstrahlung* from a laboratory X-ray source can be used for obtaining the reflectivity curve over a wide vertical momentum transfer range simultaneously, and time-resolved studies using this approach have been carried out (Paci *et al.*, 2005 ▸, 2006 ▸; Rossi Albertini *et al.*, 2003 ▸). However, the time resolution was limited to a few to several tens of minutes, partly because of the weak intensity of the *Bremsstrahlung* from the laboratory X-ray source. Using synchrotron radiation, a time resolution of seconds or less has been attained, although the measured momentum range was relatively narrow (Neissendorfer *et al.*, 1999 ▸; Bhattacharya *et al.*, 2003 ▸).

Naudon *et al.* (1989 ▸) reported a method in which the reflectivity curve over a wide vertical momentum transfer range is simultaneously measured using characteristic X-rays from a line-focus laboratory X-ray source, a knife edge close to the sample surface and a one-dimensional detector. However, with their method diffuse scattering from the incident X-ray beam cannot be separated from the signal intensity. In comparison with the conventional angle-scan method, the measured reflectivity was higher by one order of magnitude with Naudon’s method below a reflectivity of 10^−4^, owing to the overlap of the reflected beam with the diffuse scattering (Agnihotori & Ortega, 2001 ▸). A time-resolved study using Naudon’s method was carried out, although the measured reflectivity range was relatively narrow (Mizusawa & Sakurai, 2011 ▸). Other setups for quick measurement of the reflectivity curve using a Johansson-type curved crystal (Niggemeier *et al.*, 1997 ▸) or a doubly curved crystal (Chen & Gibson, 2002 ▸) have also been reported but can be expected to have the same problem as Naudon’s method.

We have previously reported a method using white synchrotron radiation from a bending magnet or tapered undulator source, a curved crystal (polychromator^1^) and a two-dimensional detector (Matsushita *et al.*, 2008 ▸, 2013 ▸; Arakawa *et al.*, 2013 ▸; Voegeli *et al.*, 2013 ▸). With this method, the whole reflectivity curve profile can be simultaneously recorded in seconds or less without mechanical motions of the sample or detector. The sample can be set horizontally, so that liquid surfaces can be measured, as was demonstrated in previous studies (Matsushita *et al.*, 2013 ▸; Arakawa *et al.*, 2013 ▸).

In this report, we present a modification of this method using characteristic X-rays from a laboratory source, which we call the convergent-beam X-ray reflectivity (CBXR) method in the simultaneous multiple-angle dispersive geometry. A convergent monochromatic X-ray beam is incident on the sample and the vertical momentum transfer continuously changes for each component of the convergent X-rays. A reflectivity curve profile in the momentum transfer range from 0.02 to 0.42 Å^−1^ was simultaneously recorded using a two-dimensional detector in 10 s. The signal intensity is separated from the diffuse scattering intensity, resulting in agreement with the conventional angle-scan method down to a reflectivity of the order of 10^−6^ with a measurement time of 100 s and down to 10^−5^ with a measurement time of 10 s. Reflectivity curves of a silicon wafer and a liquid ethylene glycol surface are shown as well. Results of time-resolved measurements with a time resolution of 10 s are also reported.

## 2. Principle of the method and the experimental arrangement   

**Figure 1:**
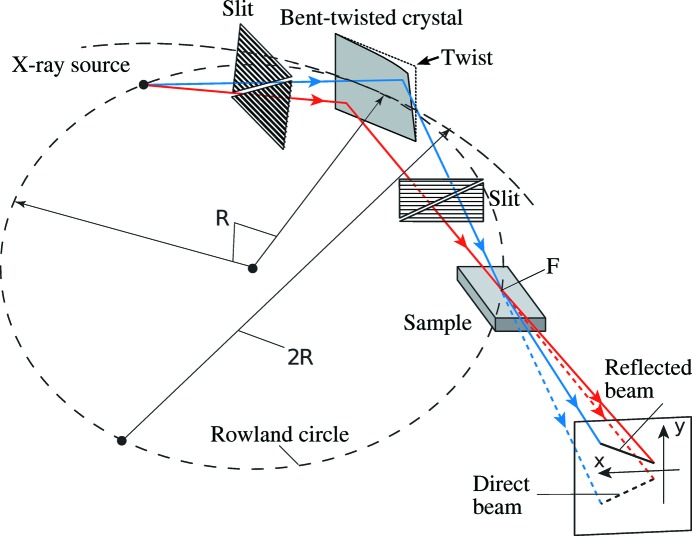
Setup of the X-ray reflectometer.

Fig. 1 ▸ shows the setup of the CBXR method. A point-focus laboratory X-ray source, the monochromator crystal and the sample are located on the Rowland circle of radius *R* (*R* = 373 mm). We used Cu *K*α_1_ radiation from a rotating anode X-ray source (Rigaku FR-D, 60 mA, 50 kV). The effective size of the X-ray source was measured as 0.1 (horizontal) × 0.3 mm (vertical) *via* a pinhole photograph.^2^ The monochromator crystal, originally a rectangular flat silicon (110) crystal (Sharan Inc., 60 × 30 × 0.5 mm), was horizontally bent with a radius of curvature of 2*R* and twisted in the vertical direction by sandwiching it between the concave and convex surfaces of a crystal bender. The silicon 220 reflection of the Cu *K*α_1_ radiation was used, giving a Bragg angle of 23.65°. The surface of the crystal can be considered as a train of tangents to a Johann–von Hamos doubly bent crystal perpendicular to the footprint. Such a bent–twisted crystal was also used in previous studies using synchrotron radiation (Matsushita *et al.*, 2013 ▸; Arakawa *et al.*, 2013 ▸).^3^ A groove was cut into the convex component of the crystal bender to secure the X-ray beam path.

Inclined fine slits were placed upstream and downstream of the monochromator crystal. The X-ray beam reflected by the crystal was horizontally focused at F. In the vertical direction, the X-ray beam passing through the inclined slits is also directed toward the point F after reflection by the crystal, because the surface normal of the crystal at the footprint of the X-ray beam was inclined downward by an amount depending on the position on the surface of the monochromator crystal. The X-ray source-to-crystal distance and the crystal-to-focus distance were both 300 mm.

**Figure 2:**
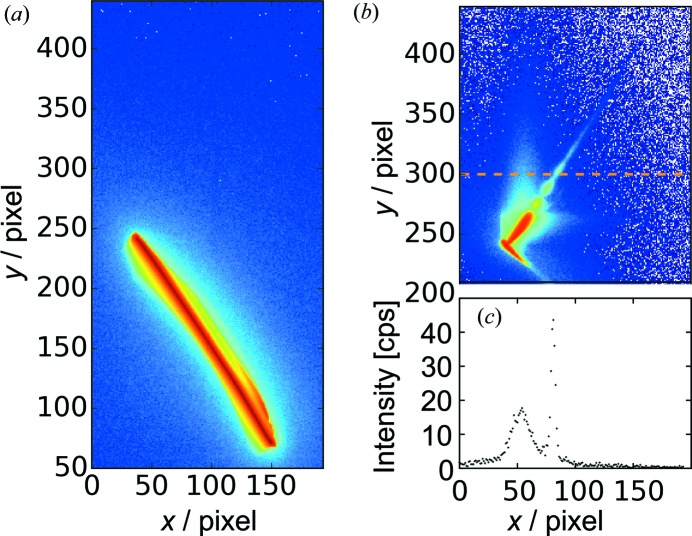
Detector images of (*a*) the direct X-ray beam (exposure time 20 s) and (*b*) the X-rays reflected from the sample (exposure time 10 s). (*c*) Intensity plot along the line *y* = 300 in (*b*).

The sample was placed horizontally at F, so that the convergent X-ray beam was incident on its surface. The glancing angle of the X-ray beam changed continuously depending on the direction of each ray, resulting in a continuous change of the vertical momentum transfer. The X-ray beam was specularly reflected by the sample. Figs. 2 ▸(*a*) and 2 ▸(*b*) show the X-ray intensity distributions *I*_0_ and *I* without and with the sample, respectively, measured by a two-dimensional detector (PILATUS 100K, DECTRIS Co. Ltd, Switzerland, pixel size 172 × 172 µm) placed 490 mm downstream of the sample. The vertical coordinate of the X-ray position on the detector is related to the vertical momentum transfer *q* by the following relations: 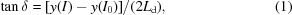


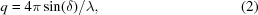
where 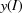 and 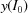 are the vertical positions of the X-ray beam on the detector surface with and without the sample, respectively, 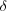 is the glancing angle of the X-rays to the sample surface, 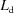 is the distance between the sample and the detector, and λ is the X-ray wavelength. Since the vertical momentum transfer of the reflected beam continuously changes on the detector surface, the distribution *I* normalized at each point along the reflected beam by the corresponding value of *I*_0_ gives the reflectivity curve. Note that the specular reflectivity can be measured without moving the sample or the detector during the measurement.

The angle γ between the beam reflected downward by the monochromator and the horizontal direction is given by 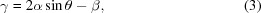
where α is the twist angle of the monochromator crystal, θ is the Bragg angle of the diffracting plane of the monochromator crystal, and β is the angle between the ray incident on the monochromator crystal and the horizontal direction. To cover the *q* range of 0.0–0.5 Å^−1^, γ should cover the range from 0 to 3.52°. The maximum twist angle was α_max_ = 8.99°. To realize this value of twisting, a careful attachment of the crystal to the bender was necessary in order to avoid breaking the crystal.

Fig. 2 ▸(*c*) shows an intensity plot along a horizontal line on the detector image, shown by a dashed line in Fig. 2 ▸(*b*). The broad peak on the left side is due to diffuse scattering of the X-ray beam components having low glancing angles. The sharp peak on the right side is from the specularly reflected X-rays, which is the signal to be recorded. The signal intensity was obtained by integrating the intensity of the right-hand peak and subtracting the background intensity as determined from fitting the intensity in the background region near the reflected beam with a straight line. With this procedure, the signal intensity was separated from the diffuse scattering intensity.

The uncertainty of the measured reflectivities was calculated from the square root of the sum of the squared statistical uncertainty of the measured intensities and the squared instrumental uncertainty. The latter arises from uncertainties in the incident angle, inhomogeneities in the detector efficiency, variations in the intensity of the incident X-rays with time, and inhomogeneities in the sample. It was assumed to be 10% of the intensity.

## 3. Reflectivity curves of test samples   

**Figure 3:**
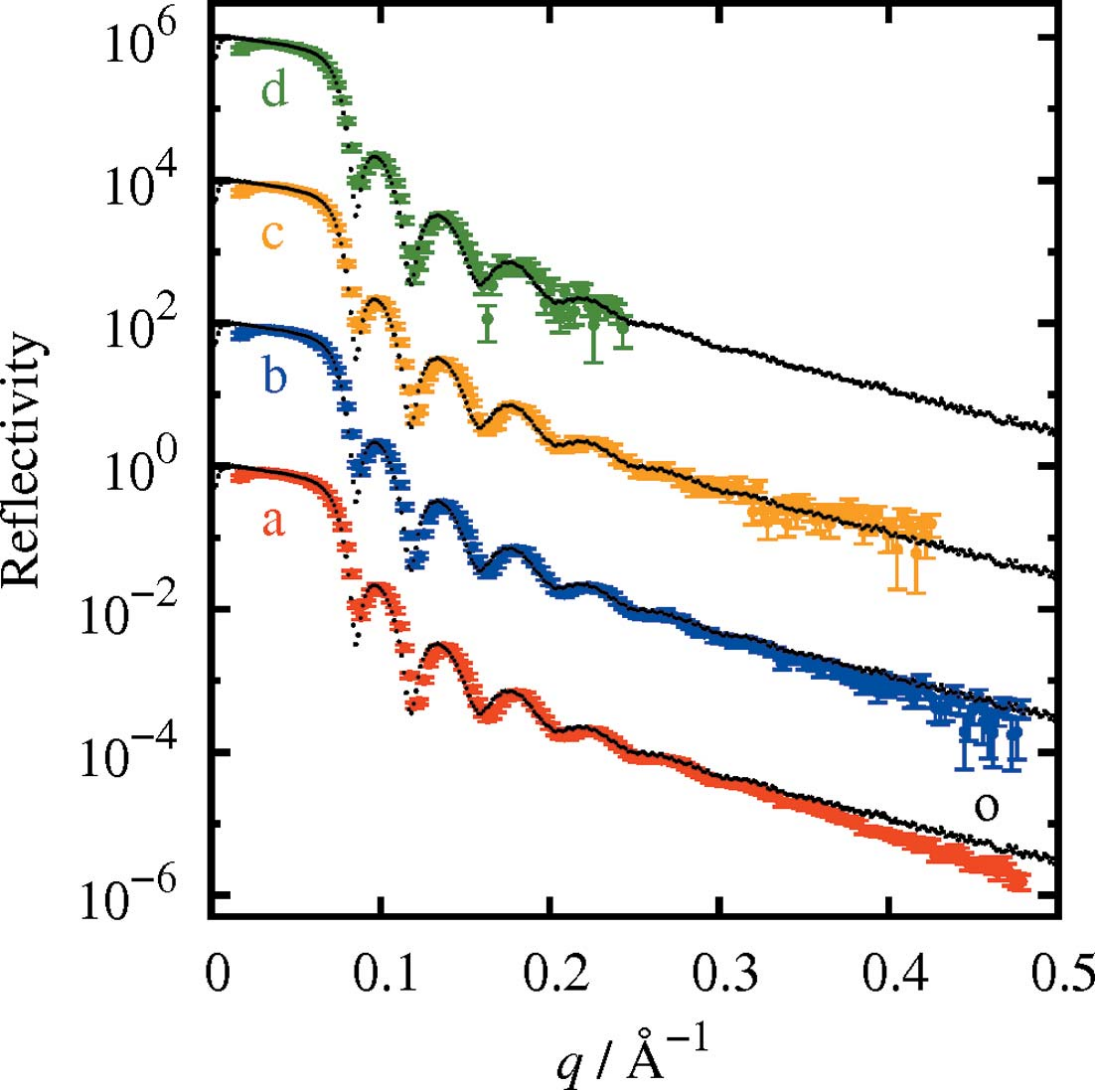
Reflectivity curves measured for a thin gold film on an SiO_2_/Si substrate. The exposure time was 1000 s for curve a, 100 s for curve b, 10 s for curve c and 1 s for curve d. b–d are each shifted two orders of magnitude along the vertical axis for clarity. Curve o was measured with the angle-scan method. Other curves shown by small black filled circles are the same as curve o, shifted for comparison with curves b–d.

Fig. 3 ▸ shows reflectivity curves measured for a thin gold film on an SiO_2_/Si substrate. Curve a (red open circles) was obtained with a measurement time of 1000 s. The reflectivity curve in the momentum transfer range from 0.02 to 0.47 Å^−1^ was obtained from one detector exposure. Curve o (small black filled circles) is the reflectivity curve measured with the angle-scan method with a measurement time of approximately 30 min. The thickness of the gold film was estimated as 13.0 nm from curve o and as 12.5 nm from curve a, using the *GenX* software (Björck & Andersson, 2007 ▸). Curves b–d were measured in 100, 10 and 1 s, respectively, with the present method. The contribution of the diffuse scattering was not subtracted in the measurement in the angle-scan mode, while it was subtracted in the measurements with the present method as explained above.

For all exposure times, Kiessig fringes were clearly observed. Even with an exposure time of 1 s, the reflectivity curve was measured down to a reflectivity of 10^−4^. The minimum measurable reflectivity was of the order of 10^−5^ and 10^−6^, for exposures of 10 and 1000 s. The agreement between the curves measured by the present method and that measured with the conventional angle-scan method is satisfactory down to a reflectivity of 10^−5^. The reflectivity measured with the present method is smaller than that measured with the angle-scan method at low reflectivities, because the background was not subtracted in the angle-scan method, while it was subtracted in the present method.

Reflectivity curves from the same gold thin-film sample measured using strong synchrotron radiation from a tapered undulator were previously reported (Voegeli *et al.*, 2013 ▸). In that case, the reflectivity down to 5 × 10^−7^ was measured in 1 s and the simultaneously measured momentum transfer range was 0.4 Å^−1^.

**Figure 4:**
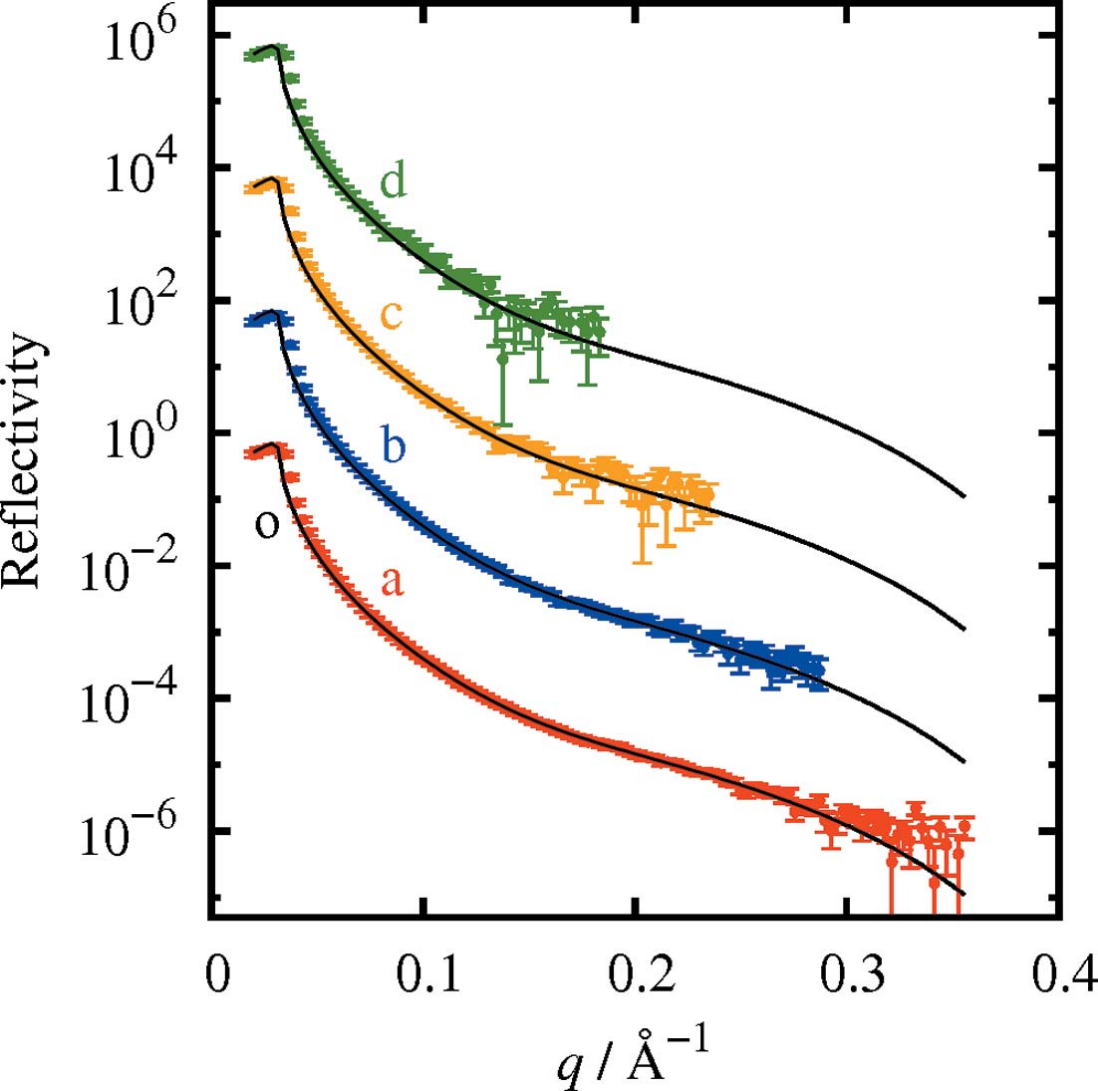
Reflectivity curves of a silicon (001) wafer. The measurement time was 1000 s for curve a, 100 s for curve b, 10 s for curve c and 1 s for curve d. b–d are each shifted two orders of magnitude along the vertical axis for clarity. The solid line o shows the reflectivity calculated from the best-fit model. Other solid lines are the same as curve o, shifted for comparison with curves b–d.

Reflectivity curves from a silicon (001) wafer were also measured (Fig. 4 ▸). For the silicon wafer, reflectivity curves were measured down to a reflectivity of 10^−5^ and 10^−6^, with an exposure time of 10 and 100 s, respectively. The measured *q* range was smaller than for the gold film, because of the lower reflectivity at high *q*.

Fitting of the reflectivity curve a of Fig. 4 ▸ to a model with an SiO_2_ film on a silicon substrate using the *GenX* software gave a density of 2.11 g cm^−3^, a thickness of 24.8 Å and a roughness of 4.7 Å for the SiO_2_ film. These are reasonable values for a silicon wafer. The reflectivity calculated from the best-fit model is shown as solid lines in Fig. 4 ▸.

**Figure 5:**
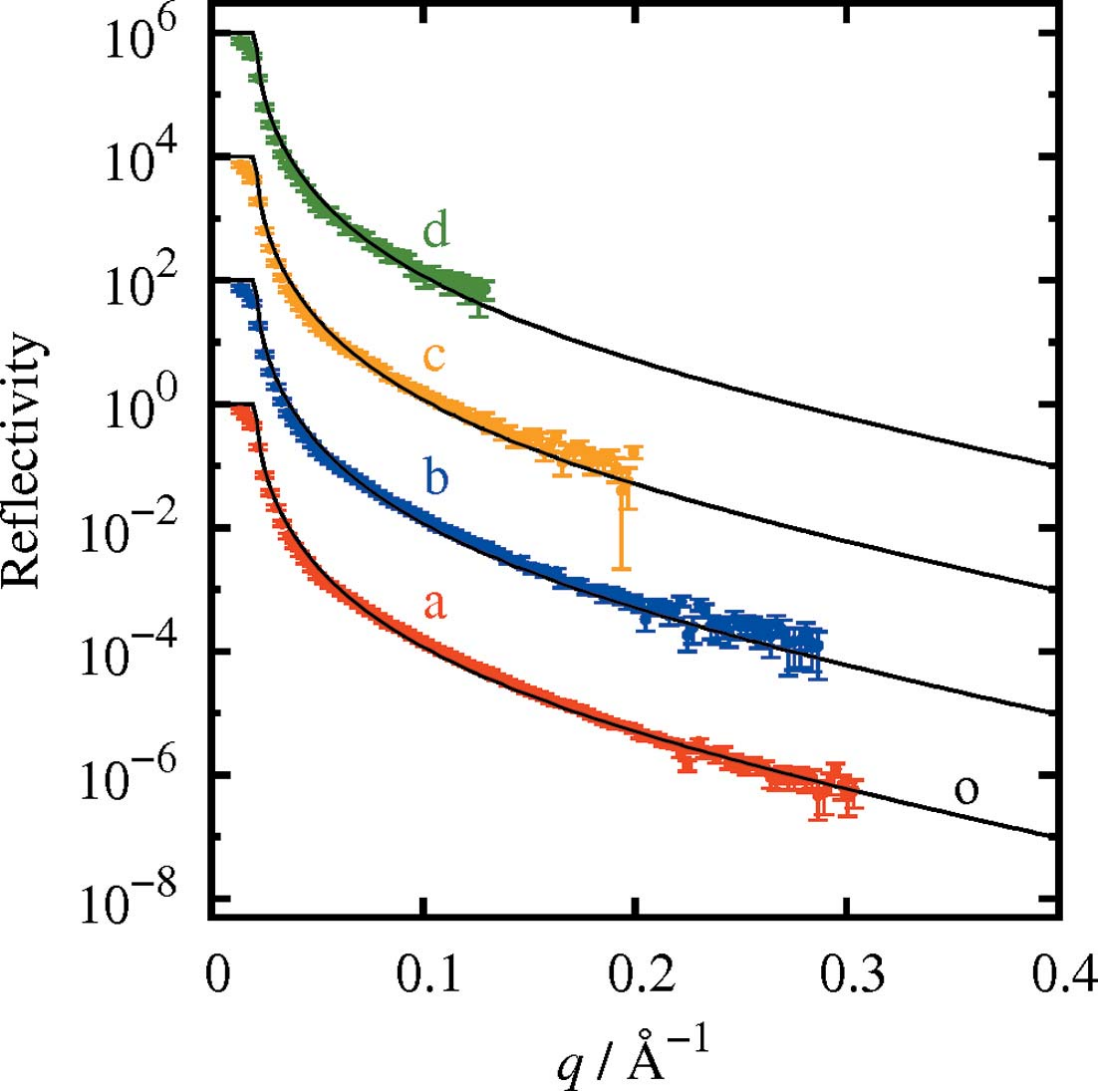
Reflectivity curves from the surface of liquid ethylene glycol. The measurement time was 1000 s for curve a, 100 s for curve b, 10 s for curve c and 1 s for curve d. b–d are each shifted two orders of magnitude along the vertical axis for clarity. The solid line o shows the calculated reflectivity. Other solid lines are the same as curve o, shifted for comparison with curves b–d.

In addition, the reflectivity from the surface of liquid ethyl­ene glycol was measured to demonstrate that the present method is also useful for liquid samples, the surface of which must always be kept horizontal during the measurement (Fig. 5 ▸). The solid lines in Fig. 5 ▸ show the calculated reflectivity obtained in the same way as reported by Yano *et al.* (2010 ▸). The discrepancy between the measured and calculated curves at small angles (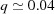
 Å^−1^) is due to the overestimation of the background in the region where it overlaps with the diffuse scattering.

## 4. Angular resolution   

The angular resolution δθ of the system depends on the following six factors: (1) the vertical size of the detector pixel element, (2) the vertical X-ray source size, (3) the vertical size of the slit aperture, (4) the spectral width of the Cu *K*α_1_ characteristic X-rays, (5) the intrinsic angular width of diffraction of the monochromator crystal, and (6) the source-to-monochromator, monochromator-to-focus and focus-to-detector distances. By taking these factors into consideration, the overall angular resolution of the present system was estimated to be 0.041°. If the signal from a single pixel element [the peak intensity of the right-hand peak in Fig. 2 ▸(*c*)] is used in the data processing, the angle resolution is 0.024°. The resulting momentum transfer resolution 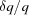 at 0.3 Å^−1^ is 0.019 Å^−1^ in the former case and 0.012 Å^−1^ in the latter case. These values are comparable to the resolution of 0.0092 Å^−1^ in the case of the synchrotron-based method (Matsushita *et al.*, 2013 ▸), but are larger than that of a conventional angle-scan reflectometer. In fact, Kiessig fringes of a 170 nm-thick TiO_2_ film on a silicon substrate were not resolved with the present reflectometer but could be observed with a conventional angle-scan reflectometer. The resolution of the present system can be improved if a smaller X-ray source and a detector with smaller pixel elements are used. For a source size of 50 × 50 µm and a pixel size of the detector of 55 × 55 µm, δθ can be estimated to be 0.009°.

## 5. Time-resolved measurement   

Time-resolved measurements were also attempted. The sample was a rutile (TiO_2_) single crystal with a (001) surface. It is known that under ultraviolet light irradiation organic contamination on the surface is decomposed and the surface becomes clean and hydro­phobic (Fujishima *et al.*, 2008 ▸). The surface of the crystal was subjected to ultra-sonic cleaning with acetone, followed by rinsing with ultra-pure water, and then contaminated by oleic acid. On the reflectometer, the surface of the sample was irradiated with UV light (385 nm low-pass filter, 470 mW cm^−2^).

**Figure 6:**
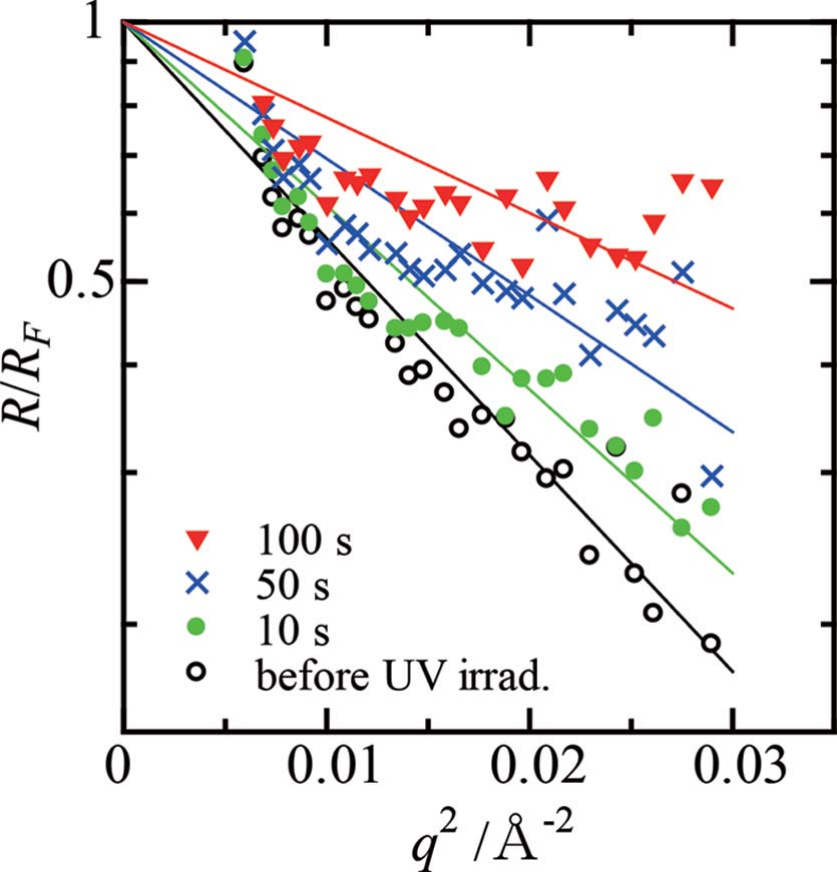
The measured reflectivity data of a TiO_2_ (001) surface contaminated with oleic acid normalized to the Fresnel reflectivity. The measurements were performed under UV light irradiation. The reflectivities measured before UV irradiation (open circles) and at 10 s (green filled circles), 50 s (blue crosses) and 100 s (red triangles) after the beginning of the UV irradiation are shown.

X-ray reflectivity measurements were performed during the UV light irradiation with an exposure time of 10 s. Fig. 6 ▸ shows the measured reflectivity data normalized to the Fresnel reflectivity of the ideal TiO_2_ interface. A significant increase in the reflectivity was observed during the UV irradiation.

**Figure 7:**
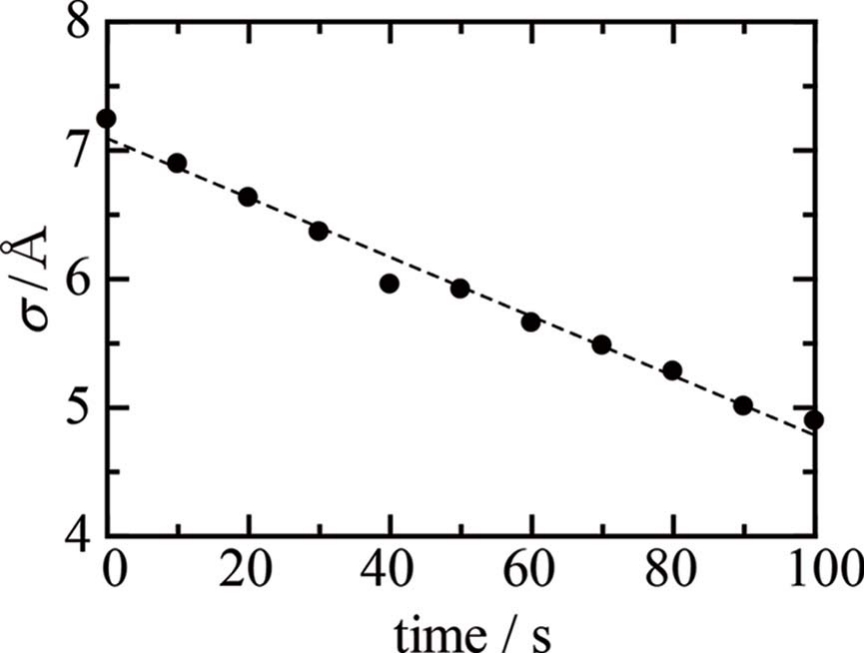
Time dependence of the surface roughness of a TiO_2_ (001) surface contaminated with oleic acid under UV light irradiation.

The reflectivity from a graded interface can be written as 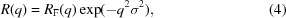
where 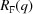 is the Fresnel reflectivity and the roughness σ is a measure of the width of the graded region (Als-Nielsen & McMorrow, 2001 ▸). The time dependence of the interface roughness estimated using equation (4) is shown in Fig. 7 ▸. The roughness of the TiO_2_ surface was reduced owing to the self-cleaning of the surface under UV light irradiation.

## 6. Summary   

In summary, we have developed an X-ray reflectometer that uses characteristic X-rays from a laboratory source and can measure the specular X-ray reflectivity curve with a measurement time of seconds to tens of seconds. Low reflectivities down to 10^−6^ can be measured accurately because the background intensity can be subtracted from the intensity of the specularly reflected X-rays. Static and time-resolved example measurements were shown.

## Footnotes

1 For the case of bending-magnet synchrotron radiation, the source is located far outside of the Rowland circle, and thus the glancing angles of the X-ray beam components change continuously along the surface of a bent–twisted crystal. X-rays with different wavelengths (energies) are diffracted at each position on the surface of the bent–twisted crystal and focused at the focus inside of the Rowland circle. We call a crystal aligned in such a geometry a polychromator crystal. In the case of the laboratory characteristic X-rays reported here, the point-like X-ray source is located on the Rowland circle. Monochromatic characteristic X-rays (with the same wavelength everywhere) are diffracted at all positions on the X-ray footprint on the crystal and focused at the focus point on the Rowland circle.

2 This was larger than the value cited by Rigaku (0.1 × 0.1 mm), probably because of misalignment of the filament position or bias voltage.

3 The main reason for using Si(220) in the present case of a conventional X-ray source rather than Si(111) used in the case of the previous work using synchrotron radiation (Matsushita *et al.*, 2013 ▸; Arakawa *et al.*, 2013 ▸) is that the twist angle necessary to realize the same glancing angle range is larger for Si(111) than for Si(220). This can be seen from equation (3). The value of β is almost zero for the synchrotron case because the X-ray source is located very far from the crystal (typically 20 m or more), while it is larger (nearly 3.8° for *q* values of 0.54 Å^−1^ in the present case) in the laboratory X-ray source case. From equation (3), the twist angle necessary to realize a certain glancing angle range is smaller if the Bragg angle θ is larger. It is difficult to twist the crystal without breaking it if the twist angle is too large. Therefore, to realize a large glancing angle range comparatively easily, we chose to use the larger Bragg angle of the Si(220) reflection.

## References

[bb1] Agnihotori, D. K. & Ortega, R. (2001). *Adv. X-ray Anal.* **44**, 302–307.

[bb2] Als-Nielsen, J., Jacquemain, D., Kjaer, K., Leveiller, F., Lahav, M. & Leiserowitz, L. (1994). *Phys. Rep.* **246**, 251–313. 10.1126/science.252.5012.1532 17834878

[bb3] Als-Nielsen, J. & McMorrow, D. (2001). *Elements of Modern X-ray Physics.* New York: Wiley.

[bb4] Arakawa, E., Voegeli, W., Matsushita, T., Yano, Y. F. & Hatano, T. J. (2013). *J. Phys. Conf. Ser.* **425**, 092002.

[bb5] Bhattacharya, M., Mukherjee, M., Sanyal, M. K., Geue, Th., Grenzer, J. & Pietsch, U. (2003). *J. Appl. Phys.* **94**, 2882–2887. 10.1103/PhysRevE.66.061801 12513309

[bb6] Björck, M. & Andersson, G. (2007). *J. Appl. Cryst.* **40**, 1174–1178.

[bb7] Chen, Z. & Gibson, W. M. (2002). *Powder Diffr.* **17**, 99–103.

[bb8] Daillant, J. & Gibaud, A. (1999). Editors. *X-ray and Neutron Reflectivity: Principles and Applications*, Lecture Notes in Physics Monographs. Berlin, Heidelberg: Springer.

[bb9] Fujishima, A., Zhang, X. & Tryk, D. (2008). *Surf. Sci. Rep.* **63**, 515–582.

[bb10] Gonzalez-Silveira, M., Rodriguez-Viejo, J., Clavaguera-Mora, M. T., Bigault, T. & Lábár, J. L. (2007). *Phys. Rev. B*, **75**, 075419.

[bb11] Matsushita, T., Arakawa, E., Voegeli, W. & Yano, Y. F. (2013). *J. Synchrotron Rad.* **20**, 80–88. 10.1107/S0909049512043415 PMC3526922 23254659

[bb12] Matsushita, T., Niwa, Y., Inada, Y., Nomura, M., Ishii, M., Sakurai, K. & Arakawa, E. (2008). *Appl. Phys. Lett.* **92**, 024103.

[bb13] Mizusawa, M. & Sakurai, K. (2011). *IOP Conf. Series Mater. Sci. Eng.* **24**, 012013.

[bb14] Naudon, A., Chihab, J., Goudeau, P. & Mimault, J. (1989). *J. Appl. Cryst.* **22**, 460–464.

[bb15] Neissendorfer, F., Pietsch, U., Brezesinski, G. & Möhwald, H. (1999). *Meas. Sci. Technol.* **10**, 354–361.

[bb16] Niggemeier, U., Lischka, K., Plotz, W. M. & Holy, V. (1997). *J. Appl. Cryst.* **30**, 905–908.

[bb17] Nüske, R., Jurgilaitis, A., Enquist, H., Dastjani Farahani, S. D., Gaudin, J., Guerin, L., Harb, M., v. Korff Schmising, C., Störmer, M., Wulff, M. & Larsson, J. (2011). *Appl. Phys. Lett.* **98**, 101909.

[bb18] Paci, B., Generosi, A., Rossi Albertini, V., Perfetti, P., de Bettignies, R., Firon, M., Leroy, J. & Sentein, C. (2005). *Appl. Phys. Lett.* **87**, 194110.

[bb19] Paci, B., Generosi, A., Rossi Albertini, V., Perfetti, P., de Bettignies, R., Leroy, J., Firon, M. & Sentein, C. (2006). *Appl. Phys. Lett.* **89**, 043507.

[bb20] Rossi Albertini, V., Generosi, A., Paci, B., Perfetti, P., Rossi, G., Capobianchi, A., Paoletti, A. M. & Caminiti, R. (2003). *Appl. Phys. Lett.* **82**, 3868–3870.

[bb21] Voegeli, W., Matsushita, T., Arakawa, E., Shirasawa, T., Takahashi, T. & Yano, Y. F. (2013). *J. Phys. Conf. Ser.* **425**, 092003.

[bb22] Yano, Y. F., Uruga, T., Tanida, H., Toyokawa, H., Terada, Y. & Yamada, H. (2010). *J. Synchrotron Rad.* **17**, 511–516. 10.1107/S0909049510013087 20567083

